# 3D mapping of static magnetic field magnitude and axial-components around a total body 3T MRI clinical scanner

**DOI:** 10.3389/fpubh.2025.1625728

**Published:** 2025-10-07

**Authors:** Francesco Girardello, Maria Antonietta D’Avanzo, Massimo Mattozzi, Victorian Michele Ferro, Giuseppe Acri, Valentina Hartwig

**Affiliations:** ^1^Institute of Clinical Physiology, CNR, Pisa, Italy; ^2^Department of Occupational and Environmental Medicine, INAIL, Rome, Italy; ^3^Department of Biomedical, Dental, and Image Sciences, University of Messina, Messina, Italy

**Keywords:** magnetic field, MRI, 3D map of magnetic field, occupational exposure, fringe field

## Abstract

**Objective:**

The technology employed in magnetic resonance imaging (MRI) systems has evolved continuously, resulting in MRI scanners with stronger static magnetic fields (SMF) B0, faster and stronger gradient magnetic fields, and more powerful radiofrequency transmission coils. The most well-known hazard associated with an MRI environment is the projectile effect due to Spatial Field Gradient (SFG). Furthermore, movement through the SFG generates a time-varying magnetic field, which in turn induces a voltage in body tissues. This has the potential to result in a range of physiological symptoms, including headache, nausea, vertigo, phosphenes, numbness, tingling, loss of proprioception, and balance disturbances.

**Approach:**

The methodology outlined in this study provides a comprehensive and reliable approach to creating a 3D map of the SMF (fringe field) around a clinical MRI facility. The methodology involves measuring the unperturbed B field, including magnitude and axial components, in specific points and subsequently performing a mathematical procedure involving fitting and interpolation.

**Main results:**

Fringe field magnitude and axial components 3D maps are presented for a 3T whole-body MRI scanner for clinical application located in a hospital facility.

**Significance:**

The map obtained could be used for a number of purposes, including the evaluation of hazard. This could be achieved by using digital tools to create a simulation of all types of MRI workers movements within the facility. The map could also be used for the training and education of MRI operators, with a view to establishing best practices. The estimation of magnetic field axial components represents a valuable enhancement, as these data can be used to calculate induced electric fields during rotational movements, such as those of the head or torso.

## 1 Introduction

Magnetic resonance imaging (MRI) is a widely utilised tool in both medical research and diagnostic imaging. In contradistinction to ionising radiation, MRI employs electromagnetic radiation, which is characterised by an energy level that is insufficient to dislodge electrons from atoms or molecules. Nevertheless, the principal hazard linked with MRI is posed by the static magnetic field (SMF), which, for the majority of clinical scanners, is constantly active (1).

The technology employed in MRI systems has evolved continuously, resulting in MRI scanner with stronger SMF (B0), faster and stronger gradient magnetic fields, and more powerful radiofrequency transmission coils (2). Many safety investigations have been carried out on 1.5T scanners, although in the last few years, many centres have installed magnets of 3.0T and above. It is imperative that all personnel involved are cognizant of and adept in the identification and mitigation of MRI hazards. Some of these hazards may include projectile accidents, whereby the powerful magnetic field produced by the MRI machine can cause metallic objects to fly into the air and possibly hurt medical professionals (3–5).

Spatial Field Gradient (SFG), is defined as the rate of change in the magnetic field as a function of position around the MRI system. The SFG is known to decrease with increasing distance from the extremities of a standard cylindrical, horizontal-field magnet and it is responsible for the attractive force on ferromagnetic objects. The SFG characterises the temporally fixed spatial gradient magnetic field surrounding the MRI system, with its regional value depending on B0 and scanner shielding (6). Passive implanted items, including vascular clips and protheses, and active implanted medical devices (AIMDs), such as pacemakers and cochlear implants, are also susceptible to forces and torques in the MRI environment, which can result in significant impairment (7–9). Consequently, AIMDs and all other medical equipment intended for use in the MRI environment are typically excluded from the 0.5 mT (5 G) fringe field. It is customary for MRI scanner manufacturers to provide a map or chart of the SMF magnitude around the scanner, also referred to as fringe field or stray field, typically in the form of an isogauss lines map, which indicates the strength of the field at specific locations. These are utilised by MRI personnel to ascertain whether the maximum field to which an implant will be subjected exceeds the “MR Conditional” value indicated on the label (10, 11). As the information is presented in different ways by each manufacturer, it is important that users understand how to interpret it for the purposes of the relevant scanner.

Furthermore, in MRI workplaces, movement through the SFG acts as a time-varying magnetic field (motion-induced TvMF) (12), inducing a voltage in electrically conductive materials, such as biological tissues, in accordance with Faraday’s law (10). Consequently, rapid body movements generate a substantial electric field within the tissue, potentially resulting in a range of physiological symptoms, including headache, nausea, vertigo, phosphenes, numbness, tingling, loss of proprioception, and balance disturbances (13). Despite the extensive literature on patient safety in MRI treatments, it is crucial to acknowledge the significant hazards faced by medical workers involved in these procedures. A variety of scientific publications exist on the occurrence of short-term sensory effects as well as on the occurrence of neurocognitive and neurobehavioural effects (14–19). However, this disturbance is typically transient. Long-term effects may include a predisposition for hypertension and sleep disturbances (20, 21). The data concerning the exposure of healthcare workers to magnetic fields during pregnancy has been determined that there are no particular deviations with regard to the duration of pregnancy, premature births, miscarriages, and birth weight (13). The paucity of epidemiological studies in this area is a key concern: there is a considerable need for high-quality data, particularly on the consequences of long-term exposure to electromagnetic fields from clinical MRI (13).

From a more technical standpoint, a number of literature studies regards the risk assessment for workers exposure to SMF and motion-induced TvMF in MRI environment. The primary objective of the exposure assessment is to verify compliance with the established exposure limits stipulated in the current regulatory framework, such as the directive issued by the European Parliament and the Council of the European Union (22) and the guidelines of the International Commission on Non-Ionizing Radiation Protection (ICNIRP) (12, 23, 24). Additionally, it is intended to characterise potential exposure scenarios within the context of epidemiological studies on MRI workers exposure. This assessment is identified as a high priority to address the existing knowledge gap concerning the associated health implications (3, 4, 25). As previously stated, the unperturbed field in the MRI environment can be determined by using the isogauss line maps provided by the scanner manufacturers (26, 27). However, given that most of the extant maps do not provide a comprehensive representation of the magnetic fields near magnets, the utilisation of isogauss line maps constitutes a rough method by which to evaluate workers’ exposure.

The magnitude of a SMF can be directly measured using commercial survey meters (28–30). However, this method is capable of providing a limited number of values for magnetic field in specific locations within the MRI room. Consequently, this approach can only provide an approximate indication of the fringe field.

A substantial number of studies have been documented in the extant literature, the objective of which was to evaluate the exposure of MRI personnel to SMFs and motion-induced TvMF in a spatially heterogeneous magnetic field (31–34). The majority of these studies were based on theoretical models or personal measurements of exposure to magnetic fields, using dosimeters. A digital tool has been presented in (35, 36) that simulates the linear path followed by an MRI worker during a routine procedure and calculates the induced electric field in a simple model of the body. The tool utilised the distribution of the fringe field, derived from the isogauss line map of a particular MRI scanner. Subsequent studies by Gurrera et al. (29) presented an analytical model based on the map of the stray field of a magnetic dipole as approximation of the magnetic field straying from a closed full-body MRI scanner. Later, they added to the model an accurate analysis of human movements: whole-body movements were recorded in a gait laboratory set up to reproduce the workspace of a room with a whole-body MRI scanner, using a stereophotogrammetric system to obtain the speed trend during the movements (37). In this study, the stray magnetic field surrounding the MRI scanner is approximated using a simple dipole model, which disregards the specific architecture and shielding of individual machines.

In this study, we present a methodology for creating a comprehensive and reliable 3D map of the fringe field of a general clinical MRI facility. The methodology employed in this study involves the measurement of the unperturbed magnetic field B in specific points, followed by a mathematical procedure involving fitting and interpolation. This process is then utilised to obtain the *B* values throughout the entire room, with a resolution of 1 × 1 × 1 cm. Unlike previous similar studies, here the map of the magnitude of *B* (|*B*|) as well as each of its axial components (*Bx*, *By*, *Bz*) is estimated. The map that has been obtained could be utilised for a number of purposes including the evaluation of hazard using digital tools to create a simulation of all types of MRI workers movements within the facility, as well as for the training and education of MRI operators with a view to establishing best practices.

## 2 Materials and methods

### 2.1 Measurement acquisition

Measurements of the magnetic field and its components in the MRI rooms were carried out using a commercial HP-01 magnetometer field analyzer (Narda Safety Test Solutions, Savona, Italy) in the area where workers typically move during their daily activities. The instrument provides a resolution of 100 nT for field strengths up to 50 mT, and 100 μT for values above 50 mT. The manufacturer specifies an accuracy of 1% for DC (direct current, i.e., static magnetic field) measurement. This level of precision is suitable for evaluating fringe fields in MRI environments, particularly when considering regulatory thresholds such as 0.5 mT and 3 mT, which are commonly used to define controlled access zones. The spatial sampling density and measurement protocol were designed to capture magnetic field variations relevant to personnel movement and to reliably detect threshold crossings.

SMF measurements were taken on a 10 × 10cm grid on planes parallel to the ground (*xz* planes) at different heights from the floor, in the area of interest (near the scanner gantry).

The measurements were taken at three different heights: *y* = 95 cm, *y* = 138 cm, and *y* = 160 cm. For a 1.70 m tall worker, these heights correspond to the genitals, torso (heart), and the head, respectively.

The cover area was the frontal area to the left of the patient’s bed starting from the MRI gantry and extending up to 110 cm along the *z*-axis (parallel to the patient’s bed) and 70 cm along the *x*-axis (perpendicular to the patient’s bed). The measurements were acquired following a procedure similar to the one described in (38). A total of 288 measurements were performed.

**Figure 1:**
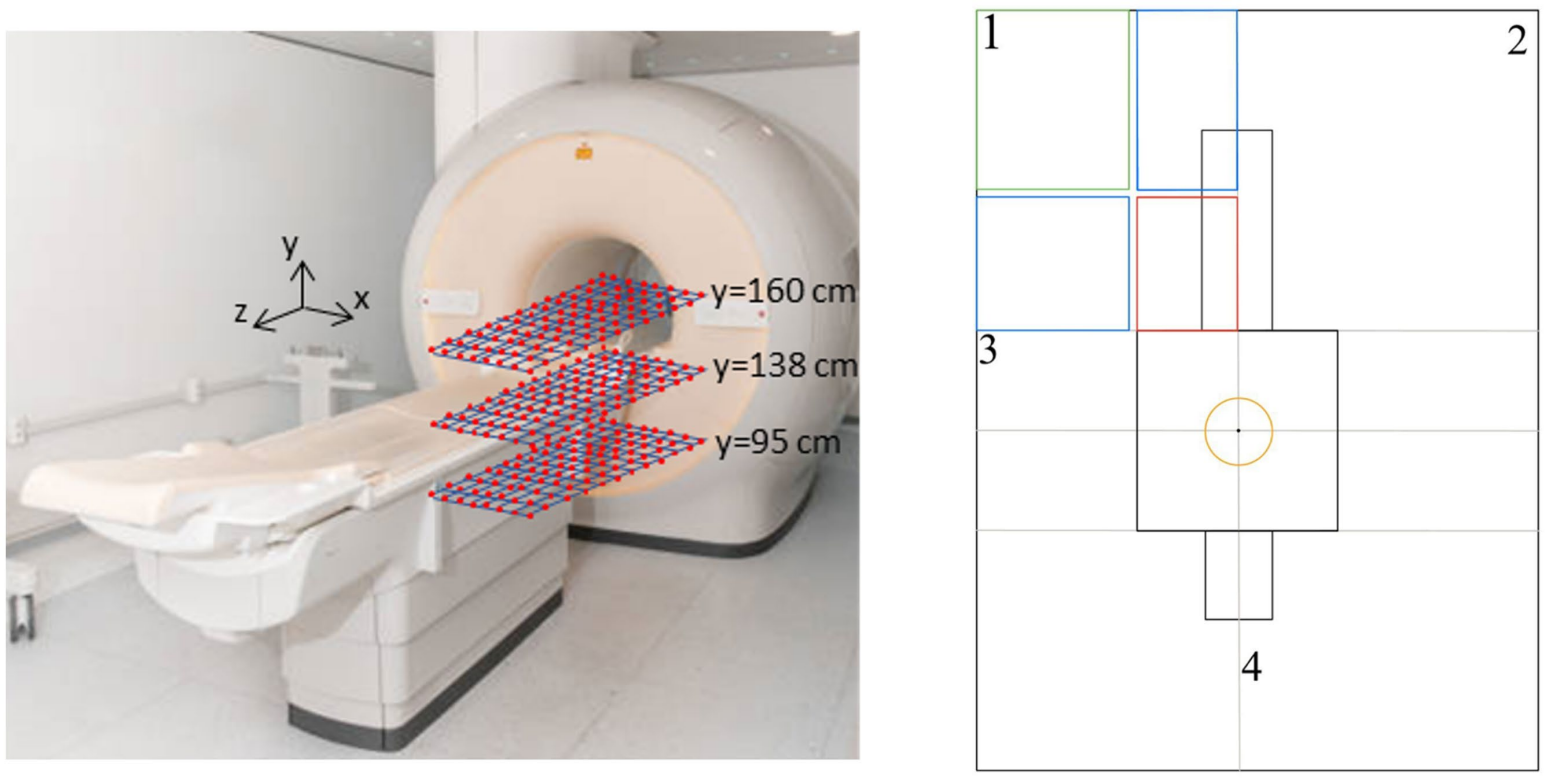
Measurement setup. Right panel: yellow circle = 3T isocentre area; red box = measurement area; blue boxes = fitting area; green box = interpolation area.

Figure 1 shows the measurement setup. The reference axes are also shown (the scanner’s isocentre has coordinate *x* = 0, *z* = 0, *y* = 105 cm). The red square represents the plane on which the measurements were taken.

### 2.2 Data processing on a single plane

All calculations described in this work were performed with homemade MATLAB®, R2020b (MathWorks, Inc., Natick, MA, USA) scripts.

As the first step in data processing, we performed quality control to eliminate potential outliers from the actual measurements. This procedure proved essential considering the extremely sensitive nature of the readings taken with the magnetometer within the experimental environment. The precision of sensor positioning is indeed critical, as even millimetre deviations from the target position can generate significant alterations in the measured values. The identification of outliers was therefore conducted following an approach based on the expected trend of values along the *x* and *z* axes, thus ensuring the integrity and reliability of the dataset used in subsequent analysis phases.

Subsequently, to create a comprehensive magnetic field map of the entire MRI room, it was necessary to extend the measurements from each plane across the entire cross-section of the room. To model the spatial distribution of the magnetic field, we implemented a parametric fitting approach using various families of non-linear functions.

The need for multiple function families was dictated by the complexity of the analysis, which required independent fitting along both the *x*-axis and *z*-axis for each of the three magnetic field components and for its magnitude. The fitting functions ranged from simple exponential combinations to more complex forms incorporating polynomial terms modulated by exponential decay.

The complexity of the functions used was progressively scaled, with models employing from 4 to 6 free parameters, in order to adequately capture both the short-range and long-range behavior of the measured magnetic field. Specifically, we explored functions that combine pure exponential terms, products of exponentials and polynomials, and hybrid forms with both linear and quadratic exponential dependencies in the spatial variable, optimizing the choice of the fitting function based on the specific characteristics of the trend to be modelled in each measurement region.

The choice of the optimal fitting functions for the experimental data was carried out through a systematic approach based on the comparison of the reduced chi-squared value. For each function the reduced chi-squared *χ*^2^_red_ was computed according to the follows equation (Equation 1):

(1) $$\chi_{\mathrm{red}}^{2}=\frac{\sum {\left(\frac{r_{i}}{\in_{i}}\right)}^{2}}{n-p}$$

where residual *r_i_* represents the difference between the measured value and the model prediction for the *i*-th point, *ε_i_* is the error associated with the *i*-th data point, *n* is the total number of experimental points, and *p* is the number of parameters in the fitting function. If *χ*^2^_red_ ≅ 1 the model describes the data well, consistent with the estimated uncertainties, if *χ*^2^_red_ << 1 the data points are too close to the model compared to their uncertainties, or the errors are overestimated, and if *χ*^2^_red_ >> 1 the model does not describe the data well, or the uncertainties are underestimated (39).

Through the previously described fitting procedures, it was possible to extend the analysis of the magnetic field to the peripheral regions (shown in blue in Figure 1) of quadrant 1 of the room. To complete the mapping of this area, an additional interpolation phase was necessary in the outermost region (highlighted in green). A function was then used to interpolate the scattered data with a natural neighbor interpolation method. This specific interpolation technique was selected following a comparative analysis with other available methods, proving to be optimal in terms of continuity and physical consistency in the magnetic field reconstruction within the region of interest (38).

The intrinsic symmetries of the magnetic field can be exploited to achieve a complete mapping of the entire space. *B* values are typically symmetrical horizontally (around the central *x*-axis), vertically (around the central *y*-axis) and radially (around the central *z*-axis). For quadrant 2, symmetry with respect to the *x*-axis was applied: the values of the magnetic field magnitude and the *By* and *Bz* components were mirrored, while the *Bx* component was inverted in sign. Similarly, using symmetry with respect to the *z*-axis, the mapping was extended to quadrant 4, this time inverting the sign of the *Bz* component.

In quadrant 3, a more sophisticated interpolation procedure was necessary, since in the lateral areas outside the scanner we did not acquire direct measurements. The procedure was based on the homogeneous region inside the scanner, indicated in the product manual, and the assumption that the realistic isolines are lines parallel to the side of the MRI magnet, in that areas. A high-resolution interpolation for 2-D gridded data in meshgrid format, using the cubic method, was iterative performed up to find a more realistic shape of the isolines in quadrant 3.

### 2.3 Uncertainty of measurement and data processing

The measurements were performed using a rigid cardboard plane on which a grid of predefined points was drawn. The plane was placed on a plastic support, allowing measurements at different heights from the floor. The reference to the scanner isocenter was obtained using the patient positioning laser, for which the distances along the *x*, *y*, and *z* axes from the isocenter are known. The probe was manually positioned on each grid point using a dedicated support to maintain it as vertical as possible and therefore perpendicular to the measurement plane. This setup ensured reproducible positioning in *x*, *y*, and *z*, with the main sources of uncertainty being:

- Variation from defined points: possible manual misplacement of the probe on the grid (estimated within a few millimeters)
- Offset from scanner isocenter: related to the accuracy of the laser reference, for which the manufacturer specifications report sub-millimeter precision
- Height positioning (*y*-axis): determined by the plastic support, with an estimated tolerance of ~3–4 mm depending on the mounting stability
- Angular alignment: minimized by the probe support, with residual tilts expected to remain below a few degrees.

The positional uncertainties were combined as a root-sum-square (RSS), and then the corresponding effect on |*B*| was obtained by multiplying this displacement by the local spatial gradient (numerically evaluated from the field map). The angular uncertainty contribution was estimated as |*B*|·(1 − cos *θ*), with *θ* the residual tilt.

The uncertainty introduced by the fitting/interpolation procedure (*σ*_fit_) was estimated by comparing the measured magnetic field magnitude ∣*B*∣ at discrete points with the corresponding values obtained from the fitted model. Specifically, for each measurement point *i*, the residual *r_i_* was calculated. The standard deviation of these residuals over all measured points provides an estimate of the absolute uncertainty introduced by the fitting procedure (Equation 2):

(2) $$\sigma_{\mathrm{fit}}=\sqrt{\frac{1}{N-1}\sum_{i=1}^{N}r_{i}^{2}}$$

where *N* is the total number of measurement points. To express this uncertainty as a percentage relative to the measured field, we calculated (Equation 3)

(3) $$U_{\text{model}}(\%)=\frac{\sigma_{\mathrm{fit}}}{B_{\text{meas}}}x100$$

where *B*_meas_ is the mean of all measured values of ∣*B*∣.

In a conservative approach, the model uncertainty was calculated by considering all measurement points, thereby accounting for the entire measured range, including low-field regions.

Finally, the total percent uncertainty *U*_tot_ was calculated as combination of all contributions using RSS method.

### 2.4 3D mapping of the MRI room

The creation of a three-dimensional map of the fringe field in the MRI room necessitates the acquisition and interpolation of a minimum of three complete planes, as previously outlined. The accuracy of three-dimensional interpolation process is directly proportional to the number of planes acquired. In order to optimise the volumetric reconstruction, the symmetry of the magnetic field with respect to the *y*-axis is exploited, thereby effectively doubling the number of planes available for interpolation. In this process, the *By* component of the magnetic field is appropriately sign-inverted, and the symmetrisation is performed with respect to the *y*-axis.

The implementation of the software has been structured according to a modular approach, which is divided into two distinct phases. The initial phase is dedicated to the management of the size data of the designated MRI room, which encompasses both the characteristic parameters of the room itself and the interpolated planes. The subsequent phase involves a general three-dimensional interpolation function that, starting from the previous data, generates the complete volumetric map.

This software architecture allows for flexible and scalable management of different MRI rooms, requiring only the specification of the reference room to generate the corresponding complete mapping. The 3D interpolation process initially involves creating a matrix containing the interpolated planes, followed by the application of volumetric interpolation using a linear interpolation method. This approach produces a 3D map of the entire room for the magnetic field magnitude, while for the individual components, it generates 3D maps of the areas in front of and behind the magnetic resonance machine. The described interpolation methodology is generalizable to other MRI rooms, provided that the experimental measurements cover a minimum surface of 70 cm × 70 cm along the *x* and *z* axes. The acquisition planes can be positioned at arbitrary heights. The presented methodology has been tested for two hospital facilities (FTGM Ospedale del Cuore—Massa and FTGM Ospedale San Cataldo—Pisa), each of which is equipped with an MRI scanner with a B0 of 3T from two different manufacturers.

## 3 Results

**Figure 2:**
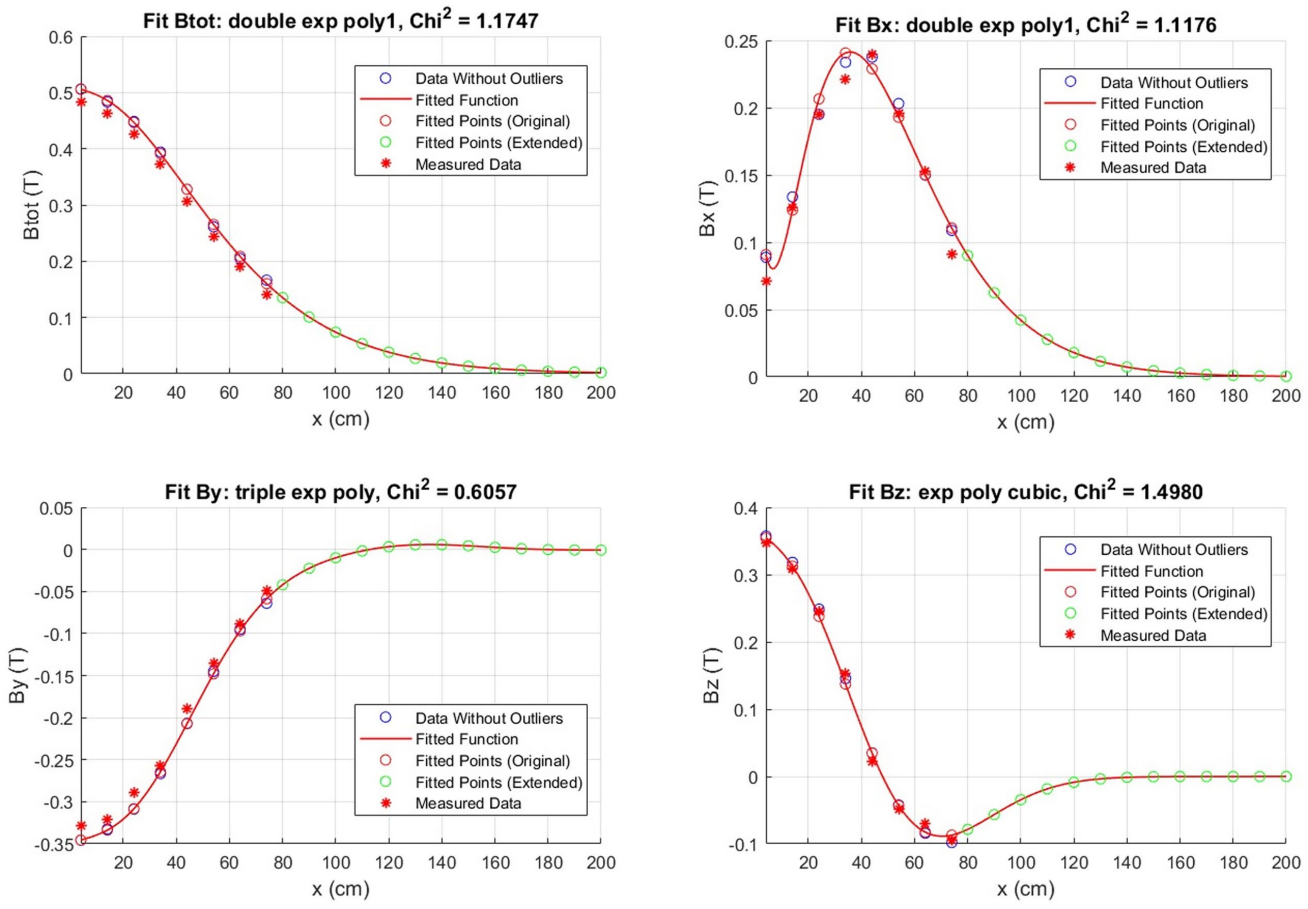
Example of a magnetic field fit obtained by the measured points throughout the room. The fit is along the *x*-axis with *z* fixed at 30 cm for the plane at *y* = 138 cm.

The results presented in this section pertain to the implementation of the aforementioned procedure on a 3T total body MRI scanner for clinical application, situated at FTGM Ospedale del Cuore—Massa, Italy. As an example, Figure 2 shows the fitting procedure for the data in terms of |*B*|, *Bx*, *By*, and *Bz* values along the *x*-axis for a fixed *z* = 30 cm from the MRI bore and *y* = 138 cm from the floor. In this particular instance, the estimated chi-squared value is equivalent to 1.174 for the magnitude, while for the components we have *Bx* = 1.117 T, *Bz* = 0.605 T, and *By* = 1.498 T.

**Figure 3:**
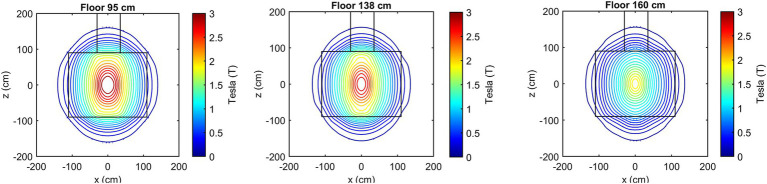
Isogauss lines reconstructed from the magnetic field data for the three reference planes at *y* = 95, 138, and 160 cm.

**Figure 4:**
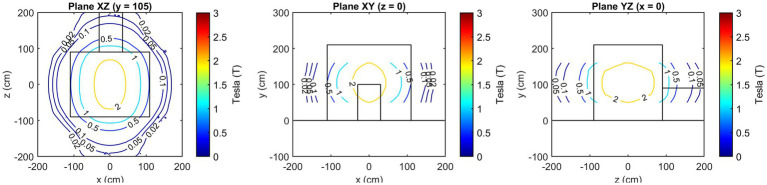
Axial, coronal, and sagittal view of the main isogauss lines for the orthogonal planes through the isocentre (@*y* = 105 cm from the floor).

Figure 3 presents the isogauss lines for the three reference planes *xz*, located at *y* = 95, 138, and 160 cm. In Figure 4, the three views (axial, coronal, sagittal) of the main isogauss lines for the orthogonal planes through the isocentre (@*y* = 105 cm from the floor) of the scanner are presented. This is the representation for the isogauss lines map that is typically provided by the scanner manufacturer.

**Figure 5:**
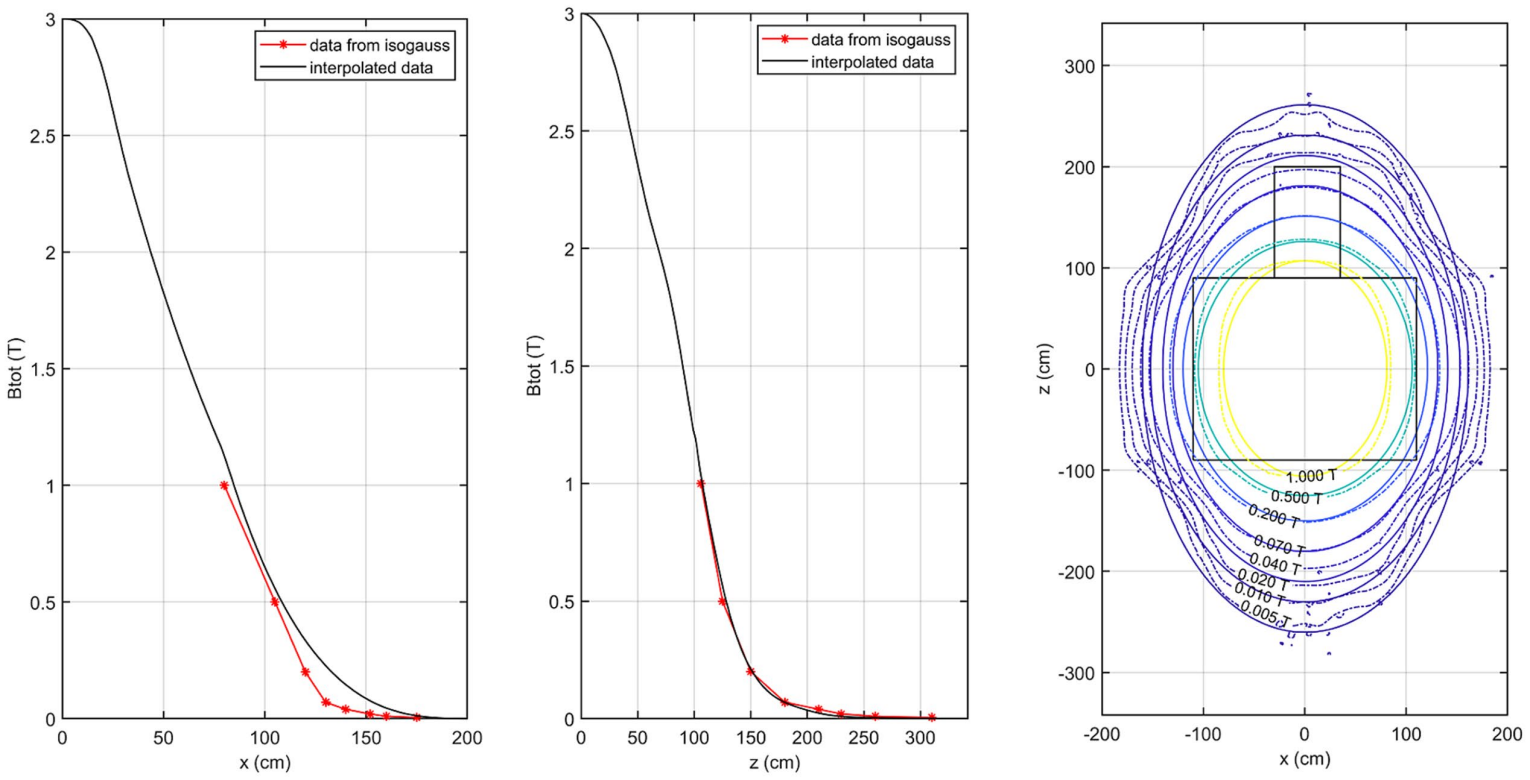
Comparison of the isogauss lines reported in the scanner manual with the estimated field data in the isocenter plane. Left panel: comparison along the *x*-axis (@*z* = 0 cm); middle panel: comparison along the *z*-axis (@*x* = 0 cm). Right panel: isogauss contours from the scanner manual overlaid with the estimated isogauss lines.

Figure 5 shows the comparison of the isogauss lines reported in the scanner manual (referenced to the plane passing through the isocenter @*y* = 105 cm) with our estimated field data in the same plane. This comparison allows direct validation of our measurements against the manufacturer-stated B0 distribution. The first panel represents the comparison along the *x*-axis (@*z* = 0 cm) while the central panel shows the comparison along the *z*-axis (@*x* = 0 cm). The right panel shows the corresponding isogauss contours from the scanner manual overlaid with the isogauss lines from our results.

**Figure 6:**
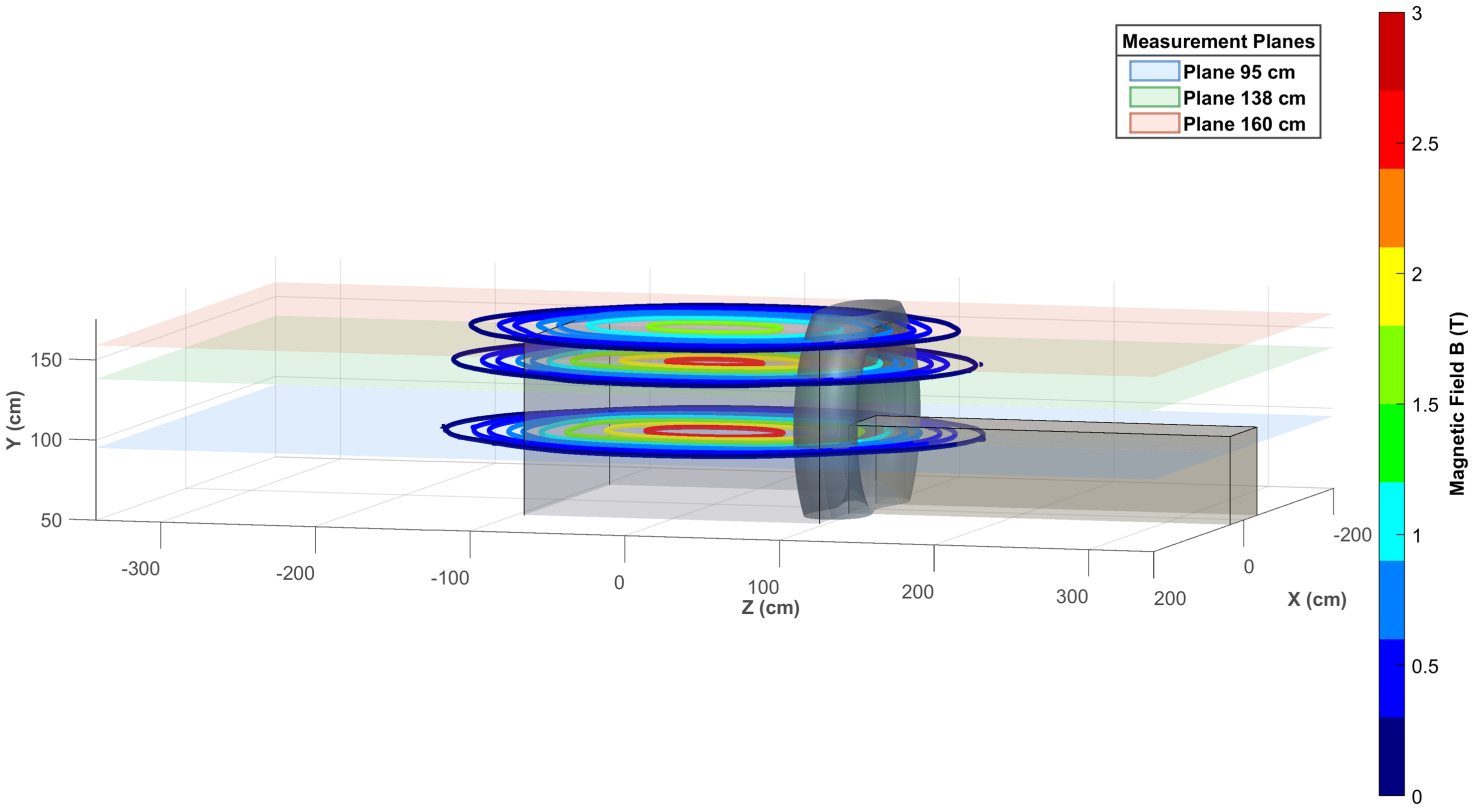
3D visual representation of the obtained magnetic field map on the three reference planes *xz*.

Figure 6 shows the 3D visual representation of the obtained SMF map on the three reference planes xz together with the scanner representation.

**Figure 7:**
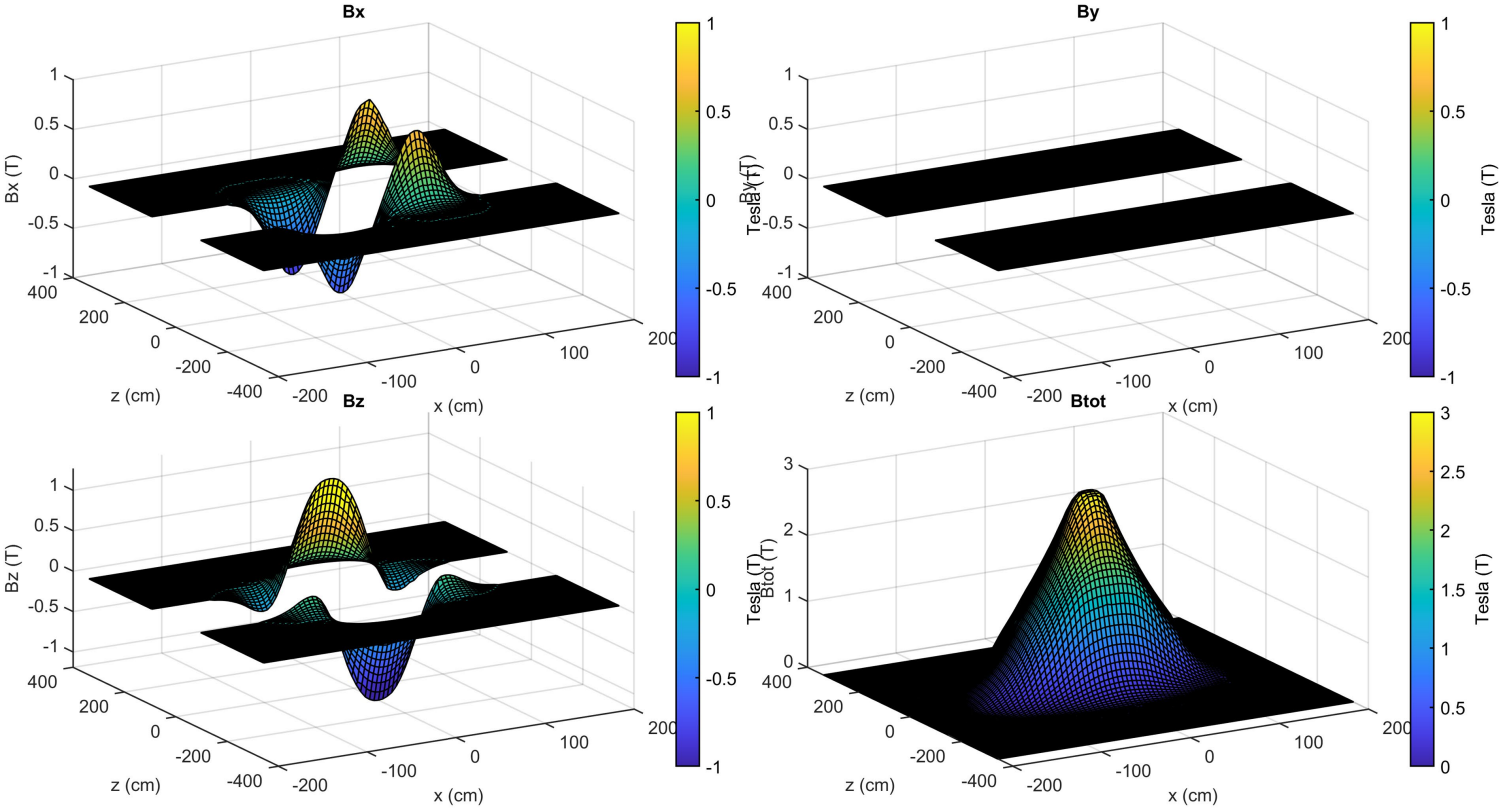
Calculated magnetic field axial components for the plane *xz* at *y* = 105 cm.

The axial components of the SMF are illustrated in Figure 7, with reference to the plane positioned at *y* = 105 cm. In contrast to the field magnitude, the components are not defined in the central part of the room, since no information is available about the magnetic field components value inside the scanner.

**Figure 8:**
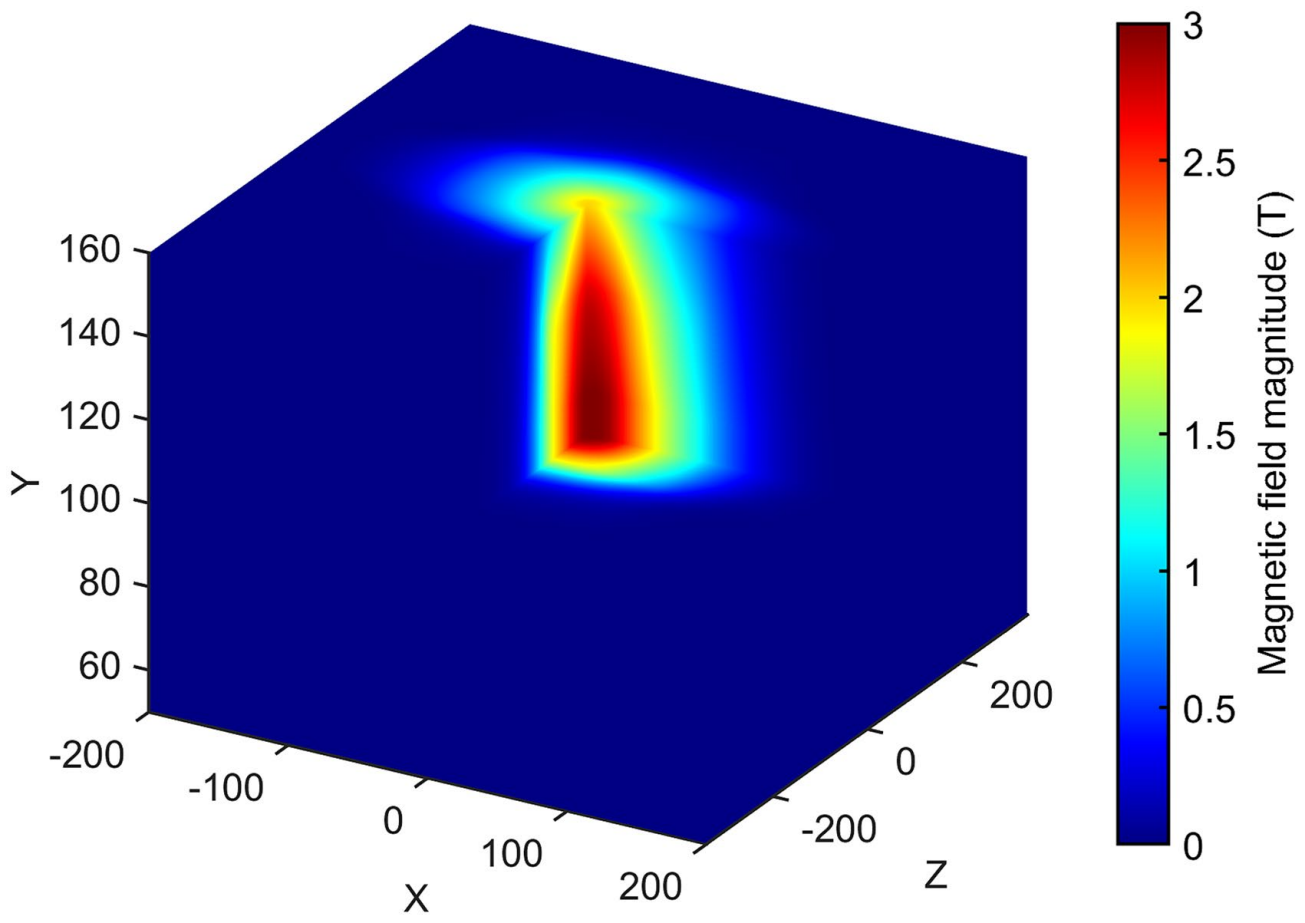
3D map of the magnetic field magnitude |B| in the entire MRI room (isocentre is at *x* = 0, *z* = 0, *y* = 105 cm). The external part has been removed to show the isocentre zone.

**Figure 9:**
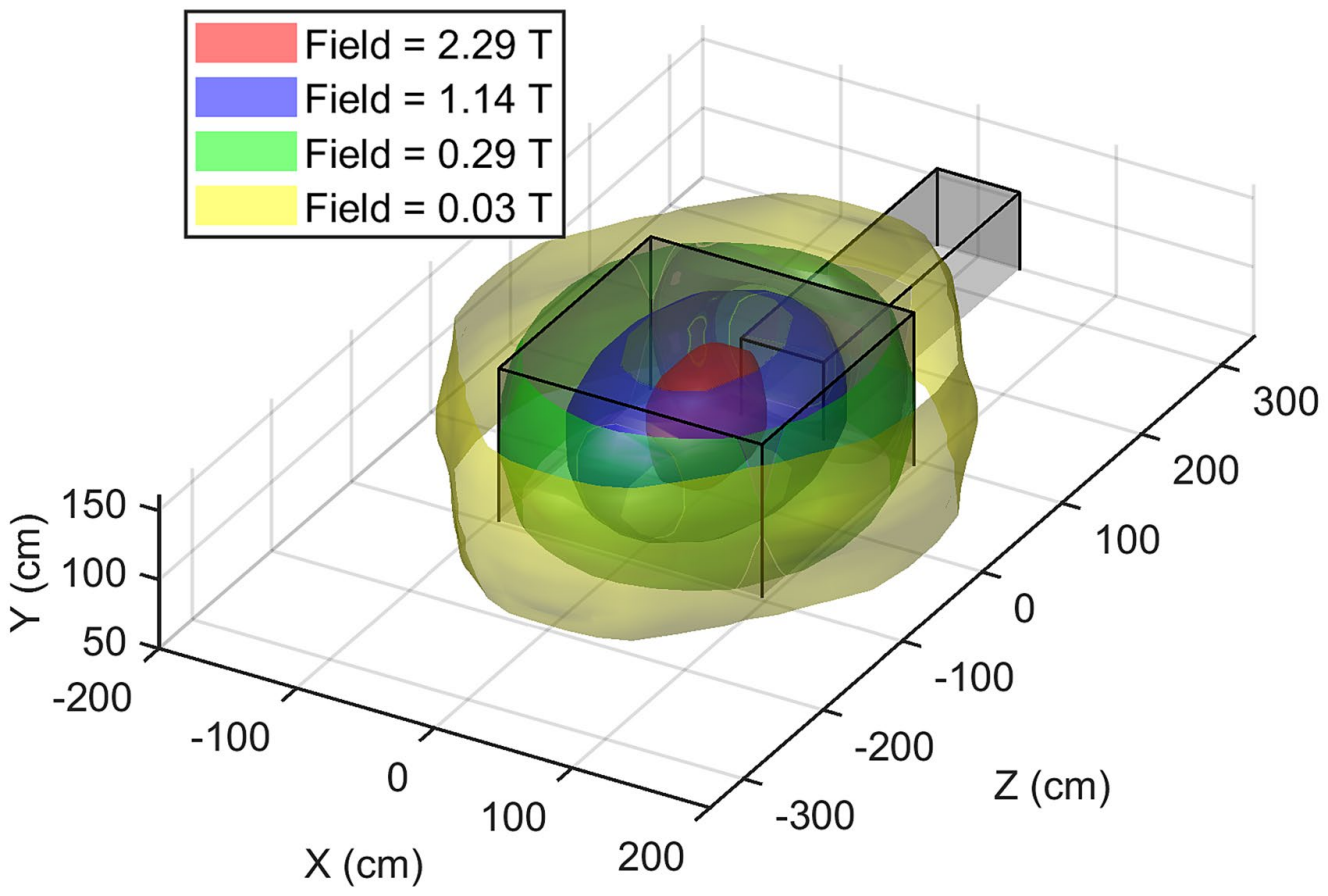
3D isogauss surfaces.

Once consistent results have been obtained for all the planes considered, spatial interpolation is performed to reconstruct the three-dimensional map of the SMF within the room. In Figure 8, the magnitude |*B*| of the SMF is plotted within a cubic volume, with its centre situated at the isocentre of the MRI scanner. Finally, Figure 9 provides a visualisation of the isogauss surface within the MRI room.

Regarding the estimation of uncertainty, Table 1 summarize the source of uncertainty with the specific estimated values used for the calculation of the total percent uncertainty.

**Table 1:** Source of uncertainty of the magnetic field map estimation.

Source of uncertainty	Estimated value
Variation from defined points	2 mm
Offset from scanner isocenter	0.5 mm
Height positioning (*y*-axis)	3.5 mm
Angular alignment	2°
Data processing uncertainty	20%

Compared to the local field value, the total uncertainty corresponds to ≈23.6% on average, with maxima up to 29.8% in the regions with lower |B| values. This shows that, while absolute uncertainties remain in the range of a few tens of mT, their relative impact is more pronounced in the peripheral regions where the field magnitude is low.

## 4 Discussion

The safety of MRI scanners is evolving with the technology and how they are used. MRI scanners with stronger SMFs (B0), faster and stronger gradient magnetic fields, and more powerful radiofrequency transmission coils are increasingly common in clinical and research settings. It is imperative that all MRI workers be aware of the potential hazards and are fully informed of the safety procedures that should be followed. Furthermore, it is essential for operators who move within the MRI room on a daily basis to be fully mindful of the fringe field present in their specific work environment. Finally, in order to proceed with the estimation of exposure to static and motion-induced TvMF, using digital simulation tools, it is first necessary to accurately estimate the 3D magnetic field distribution around a specific scanner.

It has been observed that isogauss line plans provided by manufacturers, often fail to provide a sufficiently detailed map of the fringe field. Specifically, the zone closest to the gantry, which is the one with the highest spatial gradient value, is not detailed enough (4, 38). Moreover, the isogauss line plans given by the manufacturers are generally related to the static fields in the absence of additional shielding (40).

Furthermore, the information is usually limited to the magnitude of the fringe field, with its axial components not being observed. As the fringe field is a vector quantity, it is necessary to consider *Bx*, *By*, and *Bz* in order to implement Faraday’s law in its complete form. It is imperative for the estimation of exposure in relation to electric fields induced by complex motions, such as rotation or torsion (12, 29).

Another potential methodology for obtaining a 3D map of the fringe field involves the use of mathematical modelling tools, such as simple dipole approximations (29), as previously explored in the literature. However, these models do not consider scanner-specific factors, such as active or passive shielding systems, the construction features of the installation room or the presence of nearby equipment. Consequently, 3D maps derived from these simplified models may misrepresent the actual fringe field distribution, often overestimating the field strength due to the absence of field-reducing elements. This is a particular limitation of newer MRI systems, which employ advanced active shielding technologies designed to reduce the field outside the magnet coils. These systems reduce the extent of the fringe field and generate steeper magnetic field gradients near the gantry. For all magnet types, additional passive shielding, such as strategically placed iron or high-permeability steel plates, also contributes to field shaping (6).

In this work, we propose a comprehensive methodology for reconstructing the three-dimensional map of the SMF (fringe field) around an MRI machine. The objective of the proposed approach is not to verify compliance with imposed safety limits, but rather to develop advanced educational tools for personnel operating in MRI environments. These tools will help personnel to identify and avoid higher-risk conditions and establish best operational practices.

Although the highest exposure relevance is typically associated with the zone closest to the scanner, that we identified with a red box in Figure 1, our decision to extend the field mapping beyond this region was motivated by practical and methodological considerations. In clinical environments, personnel or equipment may occasionally operate near the red-zone boundaries, where magnetic fields—though below critical thresholds—can still pose hazards, especially in the presence of active implants or ferromagnetic materials. Moreover, capturing a wider spatial distribution of the magnetic field enables a more complete characterization of field spatial gradients, which is essential for future modeling of motion-induced electric fields and exposure trajectories. Therefore, the extended mapping provides both safety-relevant and technically valuable information for more comprehensive exposure evaluations.

In this context, the following key aspects emerge from the presented work:

1. A map of the fringe field that is both detailed and reliable can be obtained through specific measurements of B in the MRI room. This is followed by mathematical fitting and interpolation procedures.
2. The methodology described herein enables the determination not only of the magnetic field magnitude but also of its individual axial components, which are essential for evaluating exposure during rotational movements of the head or torso.
3. The developed software is characterised by a modular approach, which facilitates flexible and scalable management of different MRI rooms. This approach allows for the consideration of the particular characteristics and possible complexities of the specific environment and scanner. The methodology presented has been successfully tested in two hospital facilities with 3 T MRI scanners from different manufacturers, thus demonstrating the general applicability of the approach to various clinical MRI environments.
4. The map obtained can be used for a number of purposes, including the “assessment of risks” in accordance with the Article 4 of the European Directive 2013/35/EU (22), the simulation of workers movements within the facility, and operator training. Future developments of this work will involve the use of the 3D map of the magnitude and the axial components of the fringe field to estimate the exposure of parts of the human body, such as the head or torso, during complex movements including linear and vertical displacements, rotations and flexions in a specific MRI environment.

Regarding the uncertainty budget and the practical limitations of manual probe positioning, we quantified the main sources of experimental uncertainty which then were combined with the interpolation uncertainty, conservatively set at 20% of the local |B|. The results indicate that interpolation indeed dominates the overall uncertainty in most regions, while positioning errors become relevant especially in areas of higher spatial gradients. Angular misalignment contributes less significantly but remains non-negligible in regions of stronger fields. For magnetic field evaluation for occupational exposure assessment, a total relative uncertainty of 20–30% is considered acceptable, in line with common practice for exposure evaluation in realistic workplace scenarios. Such levels of uncertainty are sufficient to reliably identify exposure thresholds and classify controlled-access zones without compromising safety-related decision-making accuracy (41, 42). Moreover, the motion induced in body electric field is generally modelled with uncertainty significantly exceeding 25% and workers motions differ even much stronger (42).

### 4.1 Limitations

A limitation of this study concerns the characterization of the probe used (HP-01, Narda). While the probe is specified as an isotropic magnetometer with three orthogonal Hall sensors, the manufacturer does not provide explicit information on the angular alignment accuracy within the housing or on the level of cross-axis coupling. As a result, when one component of the field is dominant, small parasitic signals may appear in the orthogonal channels. In the absence of dedicated calibration data, we cannot precisely quantify this contribution. However, inspection of the relative magnitude of the non-principal channels in our measurements suggests that such coupling is limited. This source of uncertainty could be addressed in future work either through manufacturer-provided specifications or through an independent calibration procedure.

Comparison with the manufacturer-provided B0 maps shows good agreement along the *z*-axis, while larger discrepancies occur along the *x*-axis especially in quadrant 3 due to the lack of direct measurements. The interpolation of the magnetic field in quadrant 3 was initially based on the assumption of a spherical homogeneous region inside the scanner, as reported in the manufacturer’s manual. However, this approach produced unrealistic isoline shapes in the lateral regions outside the bore, where no direct measurements were available. To address this, the interpolation was refined by considering a more elongated homogeneous region inside the scanner, better reflecting the actual geometry of the quasi-homogeneous field. This modification improves the realism of the interpolated distribution; however, in areas not directly covered by measurements the representation remains approximate, and further methodological improvements will be pursued in future work. On the other hand, it is well known that the manufacturer-provided isogauss maps are also approximate, since they are calculated for an ‘empty’ environment and do not consider local shielding structures or other elements present in the real measurement scenario. For this reason, the reference distribution in the manual may itself differ from the actual in-situ field.

Finally, a more detailed analysis of the uncertainty budget should be conducted also considering the propagation of uncertainty through the interpolation procedure, which is not necessarily linear for all fitting functions. A comprehensive sensitivity study would necessitate the systematic variation of each input parameter within its tolerance range, followed by the evaluation of the resulting effect on the extrapolated field maps. Such a detailed analysis is beyond the scope of the present work; however, the issue must be addressed in future studies.

## 5 Conclusion

In conclusions, the spatially detailed SMF maps produced in this study provide a solid foundation for future investigations aimed at evaluating workers exposure during standardized complex movements, such as displacement, rotation, and flexion. This direction, already supported in the literature through exposimetric studies on moving MRI workers (37, 38, 43), represents a natural and relevant extension of our current work toward more realistic, trajectory-based exposure assessments.

## Data Availability

The raw data supporting the conclusions of this article will be made available by the authors, without undue reservation.
